# Do Subjects at Clinical High Risk for Psychosis Differ from those with a Genetic High Risk? – A Systematic Review of Structural and Functional Brain Abnormalities

**DOI:** 10.2174/0929867311320030018

**Published:** 2013-01

**Authors:** R Smieskova, J Marmy, A Schmidt, K Bendfeldt, A Riecher-Rössler, M Walter, UE Lang, S Borgwardt

**Affiliations:** 1 Department of Psychiatry, University of Basel, c/o University Hospital Basel, Petersgraben 4, 4031 Basel, Switzerland; 2 Medical Image Analysis Centre, University Hospital Basel, Switzerland;

**Keywords:** Clinical high-risk for psychosis, Genetic high-risk for psychosis, At-risk mental state, functional MRI.

## Abstract

**Introduction:**

Pre-psychotic and early psychotic characteristics are investigated in the high-risk (HR) populations for psychosis. There are two different approaches based either on hereditary factors (genetic high risk, G-HR) or on the clinically manifested symptoms (clinical high risk, C-HR). Common features are an increased risk for development of psychosis and similar cognitive as well as structural and functional brain abnormalities.

**Methods:**

We reviewed the existing literature on longitudinal structural, and on functional imaging studies, which included G-HR and/or C-HR individuals for psychosis, healthy controls (HC) and/or first episode of psychosis (FEP) or schizophrenia patients (SCZ).

**Results:**

With respect to structural brain abnormalities, vulnerability to psychosis was associated with deficits in frontal, temporal, and cingulate regions in HR, with additional insular and caudate deficits in C-HR population. Furthermore, C-HR had progressive prefrontal deficits related to the transition to psychosis.

With respect to functional brain abnormalities, vulnerability to psychosis was associated with prefrontal, cingulate and middle temporal abnormalities in HR, with additional parietal, superior temporal, and insular abnormalities in C-HR population. Transition-to-psychosis related differences emphasized prefrontal, hippocampal and striatal components, more often detectable in C-HR population.

Multimodal studies directly associated psychotic symptoms displayed in altered prefrontal and hippocampal activations with striatal dopamine and thalamic glutamate functions.

**Conclusion:**

There is an evidence for similar structural and functional brain abnormalities within the whole HR population, with more pronounced deficits in the C-HR population. The most consistent evidence for abnormality in the prefrontal cortex reported in structural, functional and multimodal studies of HR population may underlie the complexity of higher cognitive functions that are impaired during HR mental state for psychosis.

## INTRODUCTION

In the last decades, schizophrenia has intensively been studied by various neuroimaging approaches (1) studying brain structural differences *(structural MRI, sMRI)* including voxel based morphometry (VBM) analysis to study whole brain differences, region of interest (ROI) approaches to study a priori selected brain regions; through (2) functional approaches to study blood oxygenation level dependent (BOLD) signal either task-related *(functional MRI*, *fMRI)*, or stimulus independent (*resting state, rs fMRI)* to study default mode network connectivity or (3) model-based assays such as *dynamic causal modeling* (DCM) to study effective connectivity.

Structurally, schizophrenia has been associated with gray matter volume (GMV) reductions in the insula, inferior and medial frontal, superior temporal, and anterior cingulate gyri, as well as in the thalamus and amygdala [1-4]. Beyond GMV, white matter volume (WMV) reductions were found in tracts that connect these structures within and between hemisphere [3]. Functionally, schizophrenia is characterized by lower activation during executive function in the dorsolateral prefrontal, anterior cingulate cortex and thalamus, and in the inferior and posterior cortical areas (see [5] for a comprehensive meta-analysis). Multimodal studies revealed structural and functional abnormalities in the insula, superior temporal, medial frontal, and anterior cingulate gyri in individuals with first episode (FE) schizophrenia [6].

The ongoing process of psychosis can be understood as a continuum of changes starting from mild cognitive impairment up to serious psychotic symptoms. Several centers worldwide are focusing on high-risk state for psychosis individuals with the aim to investigate the process. High-risk individuals can be identified according to their putative endophenotypes as genetic high-risk individuals (G-HR; [7-9]) or according to their clinical symptoms as clinical (ultra) high-risk (C-HR, UHR) or ‘at-risk mental state’ (ARMS, [10-12]) individuals. G-HR samples include monozygotic and dizygotic twins discordant for schizophrenia (non-psychotic twin) and/or subjects with at least two first- or second-degree relatives of patients affected with psychosis [13]. C-HR populations differ slightly according to the center and criteria used (for more details see [14]). The *C-HR group with first- or second-degree relatives with schizophre*nia is, in terms of clinical presentation, very similar or even overlapping with *G-HR group with present pre-psychotic symptoms*. 

In this review, we evaluate the results from longitudinal structural, as well as from functional and multimodal imaging studies with the aim to compare clinical and/or genetic high-risk individuals with controls. Part of the C-HR individuals carry inherited predisposition together with clinically manifested symptoms and C-HR individuals have shown higher transition rates as compared to the pure G-HR population. Therefore, we hypothesized to find more specific structural and functional deficits in C-HR as compared to the pure G-HR population. Furthermore, our hypothesis was to find more deficits in individuals with subsequent transition to psychosis compared to those without transition. 

Precise delineation of regions, which are abnormal in the high-risk individuals as compared to the controls, should serve as a basis for networks engaged by the selected functional paradigms. To better understand the functional abnormalities in these networks can provide deeper insight in the neurobiological mechanisms underlying the process of psychosis development.

The diameter of the circle relates schematically to the transition probability to psychosis. 

## METHODS

We systematically reviewed the neuroimaging studies on subjects at HR for psychosis focusing on similarities and differences between C-HR and G-HR compared to healthy controls (HC). While our included studies are mostly case–control studies, we adopted ‘Preferred Reporting Items for Systematic Reviews and Meta-Analyses’ (PRISMA) guidelines [16] preparing this manuscript.

### Search Strategy

We used a systematic PUBMED search to identify relevant neuroimaging publications on individuals with a high-risk state for development of psychosis. We were interested in structural MRI studies assessing whole brain (WB) or region of interest (ROI) differences during the follow-up period in high-risk individuals as compared to HC and/or first episode of psychosis (FEP) or schizophrenia patients (SCZ). We also focused on functional and multimodal neuroimaging studies in the high-risk for psychosis population. The search terms we used were as follows: high-risk, genetic high-risk, ultra high-risk, clinical high-risk, psychosis, magnetic resonance imaging, voxel-based morphometry (VBM), magnetic resonance spectroscopy, longitudinal sMRI, fMRI, neuroimaging (high risk psychosis & fMRI, high risk schizophrenia & fMRI, high risk psychosis & sMRI longitudinal, high risk schizophrenia & sMRI follow-up). Furthermore, the reference lists of the articles included in the review were manually checked for relevant studies not identified by computerized literature searching. There was no language restriction, though all included papers were in English. All included studies were published until February 2012.

### Inclusion Criteria

Studies were included according to the following criteria: (a) being an original paper in a peer-reviewed journal, (b) have enrolled subjects at G-HR for psychosis according to established criteria (see below) or subjects with C-HR and a control group or schizophrenia patients, (c) have employed structural and/or functional and/or multimodal neuroimaging methodology.

In a first step, longitudinal structural MRI studies investigating high-risk individuals for psychosis were included. For review on cross-sectional structural MRI studies, we refer to recently published reviews and meta-analyses on this issue [17, 18]. Secondly, we included functional MRI studies, and multimodal imaging approaches combining BOLD contrast with another neuroimaging technique. The included population was defined either as C-HR or as G-HR and compared with the HC group. 

### Exclusion Criteria

We excluded studies examining individuals younger than 12 years of age. Surface based morphometric studies (e.g. gyrification index or cortical thickness studies) as well as model-based studies focusing on the interpretation of the functional data and causal architecture (Dynamic causal modeling, DCM) were not included. From the studies testing primary connectivity changes between brain-regions we extracted only the BOLD differences between groups. 

### Recorded Variables

There was a battery of recorded variables extracted from each included article: characterization of the study, gender and mean age of participants according to the group, transition rate, duration of the high-risk state for psychosis (relevant in C-HR), antipsychotic medication, regional differences between relevant groups; for fMRI: cognitive domain, task, behavioral differences. Data from G-HR with present pre-psychotic symptoms (GC-HR) were listed in the table of C-HR individuals, as they fulfill both genetic and clinical criteria. Results are comprehensively reported in tables to assist the reader in forming an independent view on the following discussion.

## RESULTS

### Inclusion Criteria for Subjects at Genetic (G-HR) and Clinical (C-HR) Risk for Psychosis

The retrieved studies have defined the increased genetic risk to psychosis by including monozygotic or dizygotic healthy co-twins, siblings or children and second-degree relatives of patients affected by schizophrenic psychosis and schizophrenia spectrum disorders. The risks for developing psychosis differ across these G-HR groups with monozygotic twins having a 40–50% concordance rate for the illness over lifetime [19] while other first-degree relatives of schizophrenic patients have approximately a 10-fold increased risk compared to non-relatives over lifetime [20]

The retrieved studies have defined the increased clinical risk (C-HR) to psychosis according to the following criteria: (1) attenuated psychotic symptoms (APS) or (2) brief limited psychotic symptoms (BLIPS) or (3) genetic risk + social decline and prodromal symptoms, using Comprehensive Assessment of At-Risk Mental state (CARMS) [21] or Basel Screening Instrument for Psychosis (BSIP) [22]. Some groups have additionally added a certain combination of prodromal symptoms [10] or basic symptoms using Bonn Scale for Assessment of Basic Symptoms (BSABS) (for details see [14]).

### Data Analysis: Subjects at Genetic Risk (G-HR) and Clinical Risk (C-HR) for Psychosis

For cross-sectional structural studies we refer to recently published reviews and meta-analyses [17, 18]. It was shown that GMV reductions in prefrontal, limbic, and temporo-parietal cortex are correlates of vulnerability to psychosis [18]. Individuals with subsequent transition to psychosis had smaller GMV in prefrontal, cingulate, temporal, insular and cerebellar regions [14].

We extracted longitudinal studies comprising structural results among all high-risk groups starting with C-HR studies from the centers Melbourne, Basel, Bonn [23-29] and continuing with G-HR studies from Edinburgh and Pittsburgh [15, 30, 31] (Table **1**).

#### 1 Longitudinal Structural Neuroimaging Studies of Individuals at Genetic and/or Clinical High-Risk for Psychosis

We extracted 10 longitudinal studies following and scanning high-risk subjects after periods from 12 to 120 months. All included structural studies are listed in Table **1** according to the center where they were done. We intended to extract information about the duration of the high-risk status, antipsychotic medication and if included also data about FEP [25] or SCZ patients [28].

Three studies included the G-HR population with either no transition to psychosis [30] or with a relative low transition rate (10.5% [15] and 12% [31]). Data from overlapping C-HR studies from Melbourne showed 35% to 47% transition rate [23-26, 87]. They amended previous study by Pantelis 2003 in terms of specific regions studied by an ROI approach [24, 25] or white matter investigation [26]. In Bonn the ‘basic symptoms’ high-risk population had a 16% transition rate after 1 year [28]. The Basel C-HR population had a 50% transition rate after 4 years follow-up [29].

In the G-HR young relatives of SCZ patients showed less gray matter volume (GMV) in the orbitofrontal, anterior cingulate, and inferior frontal gyri and temporal cortex, as compared to the HC [15, 30]. Transition associated gray matter density (GMD) deficits were seen in the left inferior temporal gyrus, uncus and right cerebellum in the G-HR group [31].

C-HR compared to the HC had smaller GMV in superior temporal gyrus, insula and caudate [24, 25]; and less neuronal density as measured by N-acetyl aspartate marker (NAA) in a number of frontal and anterior cingulate regions [28]. C-HR showed transition-associated reductions in the temporal, orbitofrontal, cingulate, and cerebellar volumes [23, 29] as well as surface contraction in the right prefrontal region [87].

Summarized, G-HR and C-HR compared to the HC had volumetric abnormalities in the similar cortical areas with considerable overlap. Regions related to the tendency develop psychosis (G-HR vs. HC group) encompassed orbitofrontal, temporal, and cingulate gyri. Changes associated with prodromal symptoms (C-HR vs. HC group) showed additional deficits in the insula and caudate. More progressive reductions after transition to psychosis were found in temporal and cerebral volume in both HR groups, and in the prefrontal region in C-HR individuals additionally.

#### 2 Functional Neuroimaging Studies of Individuals at Genetic and/or Clinical High-Risk for Psychosis

For better comparison of the included populations within the same group, we extracted the functional studies, divided them according to the studied HR group into G-HR (Table **2**) and C-HR (Table **3**) studies, and presented the specific characteristics of each sample. 

##### 2.1 Functional Neuroimaging Studies of Individuals at Genetic High-Risk for Psychosis (G-HR)

Functional studies of the G-HR group [13, 32-38, 40-43, 45] are characterized in the Table **2** and results are summarized in the Table **4**. All but one [33] of the 13 included studies with G-HR were cross-sectional and mostly examined verbal memory [13, 33-35, 41, 42, 45, 68] and working memory [36, 38, 43] (Table **2**). Five studies based on an overlapping sample from the Edinburgh High Risk Study [13, 32-35], further six were conducted in the USA [36, 37, 40-43] and further two in Asia [38, 45]. We are presenting them according to the studied cognitive domain and possibly similar affected brain regions (Table **4**).

Antipsychotic medication was mostly not reported except of one study with three antipsychotic medicated HR with transition to psychosis [32]. Transition rate to psychosis among G-HR individuals was 6 and 21 % [32, 34].

Abnormalities in verbal and working memory belong to the well-known deficits in schizophrenia patients and paradigms testing these two domains are often employed to detect differences in brain function between psychotic and healthy individuals.

###### 2.1.1 Functional MRI Studies During Verbal Memory in G-HR

Hayling sentence completion task [33, 34], verbal encoding and recognition tasks [13, 35] and lexical comprehension and discrimination tasks [41, 42] were used to engage verbal memory domain, especially in language-related areas, in the inferior and medial superior frontal cortex and insula and in superior and middle temporal gyri together with thalamus, hippocampus, basal ganglia and parietal lobes. The six included studies found no behavioral differences.

Loss of lateralization in inferior frontal and superior temporal gyri and in parietal lobuli was based on reduced left hemispheric activation in the G-HR subjects, while it was due to increased right side activation in the SCZ group [41]. Compared to the HC, G-HR showed no differences [33] or activated less in language related regions [41], in inferior frontal gyri [35, 42], in the cingulate, dorsolateral prefrontal and posterior parietal cortex (especially Val/Val carriers during retrieval [13]).

Transition associated neurofunctional deficits underlying verbal memory were described in the anterior cingulate and right lingual gyri and bilateral temporal regions [34].

There is one study comparing G-HR and GC-HR for psychosis, which found differences in the parietal lobule and thalamus [13].

###### 2.1.2 Functional MRI Studies During Working Memory (WM), Attention and Social Cognition in G-HR

The word and faces N-back, spatial and auditory working memory (WM) tasks engaged predominantly prefrontal and parietal brain regions. Already G-HR subjects showed impaired performance compared to HC [36, 38, 43]. Compared to the HC the G-HR individuals showed more activation in the parietal cortex and nucleus accumbens and less activation in the parietal cortex and the cerebellum [36]. Brain activation depended on the phase of spatial WM-task, showing increased activation in the middle and superior temporal and in parietal regions during ‘encoding and maintenance’ and decreased activation in medial frontal regions during ‘retrieval’ in the G-HR group as compared to the HC group [38]. Activation in the dorsolateral prefrontal cortex was increased during the encoding phase of the spatial WM-task [38] and during 2-back task [43] in G-HR group compared to the HC.

Attention tasks as cognitive domain engaged prefrontal, temporal and parietal brain regions together with subcortical structures [37, 40]. G-HR individuals performed worse than HC in the long delay condition [37] and during the letter task [40]. 

Compared to the HC, G-HR individuals showed a stronger activation in the dorsolateral prefrontal and the middle frontal cortex [37] and in the middle temporal gyrus [40]. Additionally, they also showed less activation in a widely distributed network of prefrontal and temporal areas [40].

Social cognition engaging tasks showed activation in prefrontal, temporal and parietal cortices with amygdala activation during fear processing. Compared to the HC, G-HR showed more BOLD signal in the inferior parietal lobule and middle frontal gyrus [32]; and less BOLD signal in the temporal, insular and cingulate region and amygdala [45]. Transition associated neurofunctional deficits underlying social cognition were described in the middle frontal gyrus [32].

Taking into account slightly different brain networks engaged during described cognitive processes, we can summarize, that ‘genetic high-risk for psychosis’ was associated with functional deficits in the dorsolateral prefrontal and the cingulate cortex, the middle frontal and parietal cortex and in temporal regions. Relatively spare data about transition to psychosis among genetic high-risk subjects preclude making the conclusion about changes related to the transition to psychosis.

###### 2.1.3 Functional MRI Studies During Resting State (rs fMRI) in G-HR

Two studies investigated connectivity within default mode network (DMN) in G-HR and found contradictory results with hyperconnectivity in medial prefrontal cortex [44] and hypoconnectivity between the prefrontal and the cingulate cortex and the precuneus [39] in G-HR compared to the HC.

#### 2.2 Functional Neuroimaging Studies of Individuals at Clinical High-Risk for Psychosis (C-HR)

Functional studies of C-HR subjects [27, 32, 38, 46-59, 61-67] are characterized in the Table **3** and results are summarized in the Table 5. We included 24 functional imaging studies and divided them according to the center where the population was studied. It helps us better understand which studies had overlap and tested the same or very similar population of subjects with another functional paradigm. Most of the studies were carried out in London (9 fMRI and 6 multimodal [46-54, 61-66]), two in Aachen [58, 59], three in different centers in the USA [55-57], and in other centers in Europe and in Korea [27, 32, 38]. Two studies based on the same population were longitudinal [53, 54] and 8 included FEP group as well (4 of them were based on the same population [49-52] and 4 were not [27, 38, 57, 67]). Most of them employed WM domain and studied N-back [51, 58, 63, 64, 67], visuo-spatial [38, 50, 54], and delayed matching to sample [52] tasks. Overt verbal fluency task [46, 51, 53] investigated activity in verbal fluency domain, while ‘Theory of mind’ [27, 32]) and emotion processing task [55, 58, 59] investigated social cognition domain. Speech-associated complex paradigms engaged verbal fluency [46, 51, 53], verbal memory [47, 48] and language processing domains [56]. Executive functioning and attention cognitive domains were investigated using ‘random movement generation’ [49] and ‘visual oddball continuous task’ [57], respectively. Data from studies with more than one paradigm were extracted according to the task / cognitive domains to help the reader to better understand the complexity of abnormal processes within the underlying brain regions (Table **3** and **5**).

##### 2.2.1 Functional MRI Studies During Working Memory in C-HR 

Two studies investigated the N-back task [51, 58] and other four the visuo-spatial WM task [38, 50, 52, 54]. The phase-specific difference of WM (encoding, maintenance and retrieval of spatial stimuli) was the focus of two included studies [38, 52], while cognitive-load difference was the focus of another four studies [50, 51, 54, 58]. All WM paradigms engage WM network in prefrontal, parietal, temporal and thalamic regions.

Behaviorally, C-HR and SCZ had longer reaction times [38, 54] and made more mistakes as compared to the HC [50, 52]. Compared to the HC, the C-HR group showed less activation in the parietal lobuli, inferior frontal gyrus, insula and precuneus [51, 58] and more in the anterior insula and precentral gyrus [58] during the n-back task. Brain activation was weakest in the psychosis group, intermediate in the high-risk group and most pronounced in the HC in parietal lobule during the 1-back condition and in the prefrontal, insular and parietal cortex during the 2-back condition [51]. C-HR individuals showed an intermediary impaired activation between SCZ and HC in the dorso- und ventrolateral prefrontal cortex and in the thalamus during encoding and maintenance, respectively [38]. Additionally, C-HR showed less activation in precuneus [54] and medial frontal cortex [50] with increasing difficulty of the task compared to the HC.

The prefrontal cortex was mostly less activated in C-HR subjects as compared to the HC group during more demanding phase of WM tasks [38, 50, 51]. However, the opposite direction of changes, i.e. more activation was found in prefrontal [38] and anterio-insular region [58] as well. Very similar were the results from direct contrast of C-HR and FEP subjects. Brain activation in prefrontal cortex and insula showed a distinct vector of differences with less BOLD signal [52] as well as with more BOLD signal [50, 51] during more demanding WM paradigms. Thus, inferior and superior frontal and insular differences were involved in both risk- and psychosis-associated neurofunctional abnormalities in WM-associated network.

##### 2.2.2 Functional MRI Studies During Social Cognition Domain in C-HR

Social cognition domain studied by ‘theory of mind’ (ToM) and emotional processing tasks engaged inter alia prefrontal, medial temporal, and cingulate brain regions. During ToM paradigm, C-HR compared to the HC showed less activation in posterior cingulate and medial frontal gyrus and more activation in the frontal and temporo-parietal areas [27]. Transition-to-psychosis- and psychosis-associated abnormalities were found in the right middle frontal gyrus [32] and in the left inferior frontal and bilateral medial temporal gyri [27] respectively. 

Amygdala showed increased and prefrontal cortex decreased activation in C-HR compared to HC [55] during emotional processing. Negative emotions were associated with less activation in the insula and superior temporal, and with more activation in the cingulate and medial temporal cortex in C-HR compared to the HC [58]. Interestingly, C-HR showed more activation in response to neutral faces relative to negative ones in the inferior and superior frontal gyri, thalamus and hippocampus compared to HC [59].

##### 2.2.3 Functional MRI Studies During Verbal Fluency, Verbal Memory and Language Processing in C-HR

Brain networks activated by these paradigms encompass the frontal and temporal gyri, insula, and thalamus. There were no behavioral differences except of lower recognition accuracy in C-HR [47]. During verbal fluency tasks the C-HR compared to the HC showed less activation in the anterior cingulate gyrus [53] and more activation in the middle and superior frontal gyrus [46, 53] and insula [51]. High-risk individuals with subsequent transition to psychosis showed more activation in the superior and middle frontal gyrus, midbrain and hippocampus compared to those without transition [46]. The psychosis associated functional deficits in verbal fluency were found in the middle frontal and the precentral gyri and in the anterior insula [51].

The used verbal memory cognitive tasks including the sentence completion task, and the verbal encoding and recognition task [47, 48] focused on frontal and temporal brain regions. The C-HR had lower recognition accuracy and activated more in hippocampus during false alarm recognition [47], and less in medial and middle frontal and parahippocampal gyri during encoding, as compared to the HC [48]. More activation in the anterior cingulate cortex was found in the C-HR group, especially during response suppression compared to the HC [48].

The study by Sabb *et al.* 2010 evaluated language processing using a naturalistic task of listening and answering [56]. They found more activation in the inferior frontal, anterior cingulate, and inferior/middle temporal gyri in C-HR compared to the HC [56]. The C-HR who later transited to psychosis activated during reasoning more in temporo-frontal and striatal regions as compared to C-HR without transition [56].

Vulnerability-associated changes across above described modalities were seen mostly in a stronger activation in prefrontal, insular and cingulate regions, although this evidence was equivocal. Transition-to-psychosis related differences revealed stronger prefrontal, hippocampal and striatal activation in C-HR with transition. 

##### 2.2.4 Functional MRI Studies During Resting State (rs fMRI) in C-HR

The study by Shim *et al.* 2010 described hyperconnectivity between the posterior cingulate cortex and other seeds of DMN such as the anterior cingulate, the prefrontal and parietal cortex, and precuneus [60].

##### 2.2.5 Multimodal fMRI Studies of Individuals at Clinical High-Risk for Psychosis

Three studies investigated WM-associated brain activation in conjunction with volumetric (longitudinal [63], cross-sectional [67]) and PET [64] study. Reduced prefrontal activation was associated with GMV reduction in the same area in the ARMS subjects [63], as well as in the subgroup of ARMS subjects with longer duration of at-risk state (ARMS-LT) [67]. Altered activation in middle frontal gyrus and in parahippocampal gyrus correlated with striatal dopamine release [64]. Overt verbal fluency [61, 62] and verbal encoding and recognition [65, 66] tasks were evaluated in conjunction with additional PET and MRS data. ARMS individuals showed positive correlation between inferior frontal brain activation and pre-synaptic striatal dopamine dysfunction [61]. Additionally, prefrontal brain activation was associated negatively and temporo-hippocampal activation was associated positively with thalamic glutamate levels in high-risk individuals [62]. Hippocampal activation in ARMS, but not in HC, was positively correlated with striatal dopamine function [65] and with medial temporal glutamate levels [66].

## DISCUSSION

To our best knowledge this is the first comprehensive systematic review of structural and functional neuroimaging markers comparing subjects at clinical HR with genetic HR. We showed that genetic as well as clinical high-risk individuals for psychosis have volumetric reductions especially in prefrontal, medial temporal, and limbic brain regions compared to healthy controls. These regions, which are volumetrically reduced in schizophrenia patients, can be engaged by specific cognitive paradigms. The mostly used paradigms were working and verbal memory, and social cognition tasks, what is in line with the recently published meta-analysis of cognition in HR individuals [69]. As the results showed a high risk state for psychosis seems to be associated with widespread impairments in executive functions including social processing and memory and attention [69]. Across different modalities, we found consistent evidence for abnormal (higher and lower) prefrontal (superior, inferior, medial, or orbito- frontal gyrus) activation in high-risk individuals as compared to the HC and to the FEP groups.

We confirmed our first hypothesis with consistent structural and functional deficits in prefrontal and temporal brain regions in individuals prone to psychosis even before their transition. Similarly, prefrontal, medial temporal and subcortical regions are known to be involved in ongoing psychosis [70] and at the beginning of this disease [6]. Independent from specific brain networks activated by the selected paradigms and subsequently studied in included studies, we were primarily interested in between-group differences of healthy individuals and individuals with genetic and/or clinical high risk for psychosis. We found functional abnormalities in the prefrontal, insular, and cingulate cortex in HR individuals compared to the HC and to the FEP. Interestingly, this network is involved in switching between a task-related brain state and a brain state activated during rest period [71]. Vulnerability-to-psychosis-associated functional abnormalities across all paradigms were found in the parietal cortex, as well as in the thalamus, hippocampus, amygdala and striatum. Psychosis-associated BOLD-signal differences were found in the precuneus and in the cerebellum.

Our second hypothesis can be confirmed partially as we were not able to compare genetic and clinical high-risk individuals directly. Only one study demonstrated functional differences between G-HR and GC-HR individuals in the parietal lobule and in the thalamus [13]. Nevertheless we found more severe abnormalities in individuals with subsequent transition to psychosis compared to those without transition, namely in the middle and superior frontal gyri, hippocampus, brainstem, striatum and parietal lobe. Transition to psychosis was more often evaluated in the C-HR population, what could be related to the higher transition rate in this HR group.

Our results are in line with recently published meta-analyses and studies, which found reduced GMV in the anterior cingulate [18, 72], prefrontal [8, 73], the temporal cortex [73, 74] and in the thalamic region [72]****in the subjects prone to psychosis compared to HC. The tendency to develop psychosis, seen especially in G-HR compared to the HC group (genetic diathesis [73]), related to slightly different brain regions as compared to the changes associated with prodromal symptoms (C-HR vs. HC group). Thus, individuals with prodromal symptoms showed additional insular and caudate structural deficits. Medial temporal, insular and prefrontal cortex abnormalities seem to reflect psychosis-associated processes [18, 75] and were related to the positive psychotic symptoms [76]. We have to be aware that the above listed brain areas with structural defects are not isolated and not functioning per se during cognitive processes. They often have extensive anatomic connections to other regions, e.g. the anterior cingulate to the subcortical areas and to the dorsolateral prefrontal cortex [77]. However, prefrontal cortex (inferior, medial, and superior frontal gyri) showed reduced activation in the C-HR population compared to the HC regardless of the fMRI task used [78].

According to the recently published data, the majority of the mentioned regions are integral parts of a complex brain network, which is active during attention or executive activity and/or during resting state of the brain. It corresponds to the complexity of schizophrenia spectrum diseases unlikely to be caused just by one structural or functional deficit. Unfortunately based on extracted data we are neither able to characterize this brain network nor to specify protective or compensatory functions of the particular regions. Multimodal neuroimaging studies helps to integrate various methodologies and to improve the understanding of underlying processes. We can thus detect an association between neuro-functional and/or structural abnormalities in the prefrontal and thalamic regions and between striatal dopamine and thalamic glutamate dysfunctions. Still, more multimodal studies with longitudinal design are needed to understand relationship of involved regional and functional processes underlying the course of psychosis.

Ongoing psychosis is understood as a dynamic process, with different clinical stages [79] from the mental risk (not inevitable) state to develop psychosis, through transition to psychosis (approximately 30% of cases), its first episode and can (need not) continue to recurrent or even chronic disease. The difference between the individuals who made transition to psychosis versus high-risk individuals without transition was described as vulnerability markers of psychosis [70, 80]. Transition rate of study subjects is declining worldwide in the last years, which hampers a direct comparison of the individuals before and after the transition to psychosis [72]. Based on the population treated and followed-up in our center in Basel (FEPSY Study [10, 81]) we postulated a model with individuals with shorter and longer At Risk Mental State (ARMS-ST and ARMSL-T) with the latter still fulfilling the PACE criteria for ARMS [82]. Thus, ARMS-ST and ARMS-LT are still vulnerable to develop psychosis, but they differ in the probability to make this transition [72, 83]. We are convinced that the duration of at risk-risk state in C-HR populations plays an important role, especially in association with the protective or adaptive processes within the affected brain networks. We thus intended to extract the information about the duration of the ‘high-risk status’, namely duration from ascertainment of ‘high-risk’ until the date of MRI scans. Unfortunately, these data were mostly not presented. Allen *et al.* reported 0.1 year [48], Brüne and colleagues 0.6 year [27], Choi 4.7 years [38] of C-HR-duration. These data illustrate that the included populations can differ in their C-HR-duration and thus represent slightly different subgroups. We previously found insular GMV reduction in the ARMS-ST group compared to the ARMS-LT [83], the occurrence of deficits associated with later transition to psychosis even before such transition. These abnormalities were associated with clinical symptoms and corresponded to the previously found abnormalities associated with subsequent transition to psychosis (ARMS-T vs. ARMS-NT) [29].

## LIMITATIONS

Firstly, relatively few studies in G-HR were available, which prevented meta-analysis in some domains, and limits the robustness of imaging findings in subjects at G-HR. In particular although there have been prospective studies of subjects at G-HR, only cross-sectional studies have included functional imaging measures. Secondly, although we defined which studies were overlapping, we have not excluded these samples from our tables and reported all the results according to the cognitive domain engaged. We thus pointed out complexity of the studied subpopulations and robustness of the results. Thirdly, included functional studies were mostly cross-sectional and we were not able to evaluate differences in the brain developmental trajectory from the at risk state to the frank psychosis. Longitudinal design could help us to ascertain stages during the ongoing psychosis. Fourthly, antipsychotic medication was used in part of included studies already in the high-risk population and could have affected temporal and prefrontal GM volume [81, 84] and cortical response during cognitive functioning [85]. Fifthly, included structural studies were heterogeneous in terms of image analysis methods and relate to the difficulty of spatial normalizing, smoothing, intra- and/or inter-rater reliability of selected region, and robustness of standard parametric tests. Sixthly, functional MRI studies differed in preselected networks primary activated by the task and preclude integration of the data in quantitative analysis. Additionally, BOLD signal detects iso-energetic balance of the brain after performance of the task [86] rather than rapid physiological function of neurons. Despite of our effort to address effect of antipsychotic medication, strength of clinical symptoms, duration of follow-up and transition rates, these and other factors could influence heterogeneity across included studies.

## CONCLUSION

High-risk individuals for psychosis manifest brain functional and structural deficits. Neurostructural deficits associated with the transition to psychosis were found in the temporal and cerebellar regions in HR subjects, with additional prefrontal, medial temporal and subcortical deficits pronounced in C-HR. Neurofunctional abnormalities were studied in characteristic cognitive domains using specific paradigms. Neurofunctional deficits distinguishing high-risk individuals (both clinical and genetic) from healthy controls were found in the prefrontal, medial temporal, subcortical, and parietal and cerebellar regions. Multimodal studies help to integrate knowledge of different methodologies. There is an increasing need for longitudinal multimodal studies to understand the different stages of ongoing psychosis.

## Figures and Tables

**Fig. (1):**
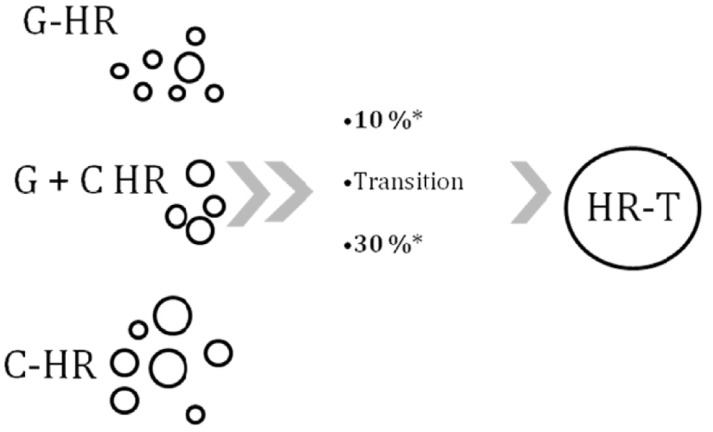
**High-risk for psychosis individuals according to their initial detection and characteristic with their probability to develop psychosis.** The diameter of the circle relates schematically to the transition probability to psychosis. * Average estimated transition rate (over a period of several years from the first diagnosis of HR state) is about 30% [12] and about 10% [15] in the of C-HR group and G-HR group, respectively. **Abbreviations:** C-HR, clinical high-risk, individuals with: attenuated psychotic symptoms (APS), brief limited psychotic symptoms (BLIPS) and trait + state factors or with ‘basic symptoms’ G-HR, genetic high-risk for psychosis, relatives of psychotic patients without psychotic symptoms G+C HR, overlap of the above mentioned groups, either G-HR with present pre-psychotic symptoms or C-HR with psychotic relatives HR-T, individuals who already transited to psychosis, can be initially either C-HR or G-HT and can subsequently develop a first episode of psychosis.

**Table 1.** Longitudinal Structural Brain Abnormalities of Individuals at Clinical and Genetic High-Risk for Psychosis as Compared to the Healthy Controls and to the Schizophrenia Patients

Center	Author Year	Overlap*	Specification	F-up (months)	HC			HR					Difference in sMRI according to the specified contrast
					N	M/F	Age	N	Subcategories	M/F	Age	Transition	
Melbourne	Sun 2009	20, Pantelis 2003	WB-CPM	12	-	-	-	35	12 C-HR-T, 23 C-HR-NT	19/16	19.9	12 (35%)	**C-HR-T vs. C-HR-NT**: greater brain surface conraction in R PF + trend in LF and occipital region
	Takahashi 2009 [25]	? Pantelis 2003 Sun 2009	ROI - GMV	12	22	12/10	22	35	12 C-HR-T, 23 C-HR-NT	19/16	19.9	12 (35%)	**C-HR-T and FEP < HC and/or C-HR-NT**: STG and caudate
	Takahashi 2009 [24]	31 and 16 Sun 2009	ROI - GMV	12 - 48	20	12/8	21.6	31	11C HR-T, 20 C-HR-NT	17/14		11 (35.5%)	**C-HR-T < C-HR-NT or HC**: bil insula
	Walterfang 2008 [26]	20, Pantelis 2003	WB - VBM- WMV	12	.	.	.	21	10 C-HR-T, 11 C-HR-NT	n.a.	19.7	10 (47.6%)	**C-HR-T < C-HR-NT**: WMV in the L fronto-orbital fasciculus, R optic radiation and inferior cerebellum
	Pantelis 2003 [23]	.	WB -VBM	12	.	.	.	21	10 C-HR-T, 11 C-HR-NT	n.a.	19.7	10 (47.6%)	**C-HR-T < C-HR-NT**: in L parahippocampal, fusiform, OFC, CC and cerebellum
Basel	Borgwardt 2008 [29]	.	WB - VBM	36 - 48	.	.	.	20	10 C-HR-T, 10 CHR-NT	12/8	24.7	10 (50%)	**C-HR-T < C-HR-NT**: in OFG, SFG, ITG, medial and superior ParC and cerebellum
Bonn	Jessen 2006 [28]	.	VOI - MRS	11.6 (SD 7.55)	31	.	.	19	10 EHR 9 LHR	EHR: 5/5 LHR: 4/5	EHR: 27.0 LHR: 28.7	3 HR-T (16%: 2 of EHR, 1 of LHR)	**C-HR and SCZ < HC**: NAA/Cr and NAA/Cho in L frontal lobe. C-HR** and SCZ < HC**: NAA/Cr in ACG; C-HR**-T < C-HR-NT**: NAA/Cho in ACG; HR-T > HR-NT: Cho/Cr in ACG, no diff in NAA/Cr
Edinburgh	McIntosh 2011 [15]	Job 2005	ROI	120	36			162	G-HR			17 (10.5%)	**G-HR < HC**: WBV and bil PF and temporal lobes
	Job 2005 [31]	.	WB - VBM - GMD	28	19	12/7	21	65	47 G-HR-, 18 GC-HR	34/31	21.4	8 (12%)	**G-HR-T < G-HR-NT**: GMD in L ITG, L uncus, R cerebellum
Pittsburgh	Bhojraj TS 2011 [30]	.	WB	12	27	11/16	16.6	23	G-HR	12/11	15.4	0	**G-HR < HC**: in bil OFG, L AC, L medial PF, R IFG, L TL

**Abbreviations:** AC, anterior cingulate; ACG, anterior cingulate gyrus; bil, bilateral; CC, cingulate cortex; Cho, choline; Cr, creatine; CPM, cortical pattern matching; EHR, early high risk (basic symptoms); f-up, follow-up; GC-HR, G-HR with present pre-psychotic symptoms; G-HR, genetic high-risk; G-HR-NT, G-HR without transition to psychosis; G-HRT, G-HR with transition to psychosis; GMD, gray matter density; GMV, gray matter volume; HC, healthy controls; FG, inferior frontal gyrus; ITG, inferior temporal gyrus; M/F, male/female; N, number of individuals, L, left; LHR, late high risk (correspond to the C-HR); MRS, magnetic resonance spectroscopy; NAA, N-acetyaspartate OFG, orbito-frontal gyrus; ParC, parietal cortex; PF, prefrontal; R, right; ROI, region of interest; SCZ, schizophrenia patients; SFG, superior frontal gyrus; STG, superior temporal gyrus; TL, temporal lobe; VBM, voxel based morphometry; WBV, whole brain volume; VOI, volume of interest; WB, whole brain; WMV, white matter volume; ., missing data

* N of overlapping subjects & author of the overlapping study

**Table 2.** Overview of Functional Studies of Individuals at Genetic High-Risk for Psychosis

Center	Author Year	Specification			HC characterization			GHR characterization		
		Study	Cognitive domain	Task	N	M/F	Age years (SD)	N	M/F	Age years (SD)
Edinburgh	Baig 2010 [13 ] **	C-s	VM	Verbal encoding and recognition task	19 (Met carriers)	8/11	26.9 (2.9)	39 (Val/Val)	15/4	25.91 (3.43)
	Marjoram 2006 [32 ]	C-s	SC	ToM	13	8/5	29.6 (1.6)	24 (12 GC-HR)	GC-HR: 5/7 G-HR 8/4	G-HR: 30.8 (2.1) GC-HR: 28.9 (1.6)
	Whalley 2009 [33 ] *	Long (18 months)	VM	HSCT	16	10/5	26.1 (2.2)	61 (32 NN, 9 NP, 15 PN, 5 PP)	28/33	NN 27.5 (2.4), NP 25.0 (2.6), PN 25.4 (3.3), PP 27.3 (2.8)
	Whalley 2006 [34 ]	C-s	VM / EF	Sentence completion	21	13/8	26.8 (2.7)	66 (21 GC-HR, 4 HR-T)	29/37	G-HR 27.0 (3.2), GC-HR 25.5 (3.1), HR-T 22.8 (4.5)
	Whyte 2006 [35 ]	C-s	VM	Verbal classification and recognition	21	13/8	26.8 (2.7)	68 (27 GC-HR)	31/37	G-HR 26.6 (3.3) GC-HR 25.1 (3.1)
St. Louis, Missouri	Brahmbhatt 2006 [36 ] **	C-s	WM	Word and face n-back task	72	30/42	20.3 (3.5)	18	7/11	20.7 (4.0)
	Delawalla 2008 [37 ]	C-s	AT	AX-CPT (variation of the continuous performance)	92	39/53	20.2 (3.4)	30	14/16	21.3 (3.5)
Seoul	Choi 2011 [38 ]**	C-s	WM	Spatial WM	16	9/7	21.37 (2.28)	38 (21 UHR)	G-HR: 9/8 UHR: 12/9	G-HR: 20.71 (5.50) UHR: 21.62 (4.08)
	Jang 2011 [39 ] **	C-s	DMN	rs fMRI	16	9/7	22.06 (1.65)	16	9/7	21.32 (5.65)
New Mexico	Filbey 2008 [40 ]	C-s	AT	Attention	8	5/3	41 (18-60)	6 (POC)	2/4	53 (49-59)
New York	Li 2007 [41 ]	C-s	VM	Visual lexical discrimination	36	17/9	22.9 (5.4)	21	7/14	20.1 (4.3)
Michigan	Rajarethinam 2011 [42 ]	C-s	VM	Lexical comprehension task	17	9/8	14.5 (3.5)	15	7/8	15.9 (3.1)
Boston	Seidman 2006 [43 ]	C-s	WM	Auditory WM + visual 2-back	24	10/14	18.1 (3.3)	21	12/9	19.9 (4.0)
Massachusetts	Whitfield-Gabrieli 2009 [44 ] ***	C-s	WM + DMN	N-back relative to rs fMRI	13	.	.	13	.	.
Bangalore	Venkatasubramanian 2010 [45 ]	C-s	SC	Facial emotion recognition	16	14/2	24.4 (3.7)	17	14/3	25.2 (4.2)

**Abbreviations:** AT, attention; V, vigilance; C-HR, clinical high risk; C-s, cross-sectional; DMN, default mode network; EF, executive functioning; HC, healthy control; HR, highrisk; HR-T, high-risk with transition;

* HR NN, HR asymptomatic at both time points. HR NP, went from asymptomatic to symptomatic. HR PN, went from symptomatic to asymptomatic. HR PP, symptomatic at both time points [33]; HSCT, Hayling sentence completion task; G-HR, genetic high risk; GC-HR, G-HR with present pre-psychotic symptoms [32, 34]; G-HR-, never any partial or transient symptoms = G-HR [32, 34]; Log, longitudinal; M/F, male/female; N, number of individuals, L, POC, presumed obligate carriers; rs fMRI, resting state fMRI; SC, social cognition; ToM, "Theory of mind"; VL&M, verbal learning and memory; WM, working memory

** Clinical outcomes were reported only by 4 studies as following: PANSS general total: HC 19.37 (SD 4.42) HR 21.15 (SD 6.00 [13]); SAPS global score: HC 0.03 (SD 0.10), SCHIZ 1.55 (SD 0.59), HR 0.09 (SD 0.19) SANS global score: HC 0.17 (SD 0.30), SCHIZ 2.00 (SD 0.81), HR 0.43 (SD 0.64 [36]); PANSS total UHR 54.00 (SD 13.54) PANSS total SCZ 55.00 (SD 14.43 [38]); and PANSS total G-HR 32.88 (SD 3.72) [39] Only 3 studies reported also data from schizophrenia patients treated with antipsychotics: Brahmbhatt 2006 with 19 [36], Choi 2011 with 15 [38] and Li 2007 with 20 patients [41].

*** groups were matched with no other details published + correlation with SAPS score and connectivity was assessed with no raw data published

**Table 3.** Overview of Functional Studies of Individuals at Clinical High-Risk for Psychosis

Center	Author Year	Specification			HC characterization			C-HR characterization				
		Study (Overlap)	Cognitive domain	Task	N	M/F	Age years (SD)	N & characteristic	M/F	Age years (SD)	APS Y/N (N)	PANSS total
London	Allen 2012 [46]	C-s + subgroup PET	VF	Overt verbal fluency	24	16/8	25.5 (4.4)	41 (34 UHR-NT / 7 UHR-T)	UHR-NT: 22/12 UHR-T: 5/2	UHR-NT: 24.44 (5.28) UHR-T: 23.11 (3.12)	Y (4)	UHR-NT: 47.66 (SD 11.8) UHR-T: 50.17 (SD 13.16)
	Allen 2011a [47]	C-s	VM	Verbal encoding and recognition	22	14/8	27.6 (1.6)	18 ARMS	10/8	27.10 (4.95)	Y (2)	ARMS 45 (SD 13)
	Allen 2010 [48]	C-s (Allen 2011)	VM	HSCT	15	8/7	25.8 (45.0)	15 ARMS	9/6	26.85 (4.95)	Y (2)	ARMS 47 (SD 13)
	Broome 2010a [49]	C-s (Broome 2009)	EF	Random movement generation	15	11/4	25.4 (4.9)	17 ARMS	12/5	24.2 (4.1)	AN	ARMS 51.9 (SD 12.7)
	Broome 2010b [50]	C-s (Broome 2009)	WM	PAL (spatial WM)	15	11/4	25.4 (4.9)	17 ARMS	12/5	24.2 (4.1)	AN	ARMS 51.9 (SD 12.7)
	Broome 2009 [51]	C-s	WM / VF	N-back, Overt verbal fluency	15	11/4	25.4 (4.9)	17 ARMS	12/5	24.2 (4.1)	AN	ARMS 51.9 (SD 12.7)
	Benetti 2009 [52]	C-s (Broome 2009)	WM	DMTS	14	9/5	26.0 (4.6)	16 ARMS	10/6	24.13 (3.97)	AN	ARMS 44.2 (SD 10.9)
	Fusar-Poli 2011a [53]	Long (Fusar-Poli 2010a)	VF	Overt verbal fluency	15	9/6	25.2 (5.1)	15 ARMS	8/7	24.36 (4.48)	Y (7)	bl: 46.57 (SD 12.09), f-u: 42.86 (SD 15.29)
	Fusar-Poli 2010a [54]	Long	WM	PAL (visuospatial)	15	9/6	25.2 (5.1)	15 ARMS	8/7	24.36 (4.48)	Y (7)	bl: 46.57 (SD 12.09), f-u: 42.86 (SD 15.29)
Los Angeles, USA	Gee 2012 [55]	C-s	SC	Emotion processing	14	5M 7F	18.7 (2.5)	20 C-HR	10/10	18.8 (2.4)	Y (5)	.
	Sabb 2010 [56]	C-s	Language processing	Naturalistic discourse processing paradigm	24	12M 12F	18.5 (3.2)	40 C-HR	28/12	C-HR-NT 16.8 (3.1) / C-HR-T 18.4 (4.2)	Y (7)	.
North Carolina, USA	Morey 2005 [57]	C-s	AT	Visual oddball continuous performance	16	9M 7F	28.0 (11.6)	10 UHR	5/5	22.6 (4.4)	Y (2)	UHR: 41.7 (SD 12.1)
Aachen	Pauly 2010 [58]	C-s	WM + SC	n-back + negative vs. neutral olfactory stimulation	12	.	24.5 (4.7)	12 C-HR	10/2	24.22 (4.61)	Y (5)	PANSS general: C-HR 26.33 (SD 8.46)
	Seiferth 2008 [59]	C-s	SC	Emotion discrimination	12	10M 2F	24.5 (5.0)	12 C-HR	10/2	25.5 (4.6)	Y (4)	PANSS global: C-HR 52.2 (SD 15.5), PANSS pos: C-HR 11.8 (SD 3.9) PANSS neg: C-HR 14.0 (4.8)
Seoul	Choi 2011 [38]	C-s	WM	Spatial WM	16	9M 7F	21.4 (2.3)	38 UHR	12/9	21.62 (4.08)	Y (5)	UHR 54.00 (SD 13.54)
	Shim 2010 [60]	C-s	DMN	rs fMRI	20	11/9	21.7 (2.1)	19	11/8	20.8 (4.1)	Y (3)	57.4 (SD 14.6)
Edinburgh	Marjoram 2006 [32]	C-s	SC	ToM	13	8/5	29.6 (1.6)	24 12 GC-HR (HR+)	GC-HR: 5/7	GC-HR: 28.9 (1.6)	.	.
Bochum	Brüne 2011 [27]	C-s	SC	ToM	25	16M 9F	28.8 (4.1)	10 PROD	7/3	25.5 (5.3)	Y (3)	PANSS pos. PROD 12.1 (SD 2.9), PANSS neg PROD 14.5 (SD 4.1)
London	Fusar-Poli 2011b [61]	fMRI + PET - C-s	VF	Overt verbal fluency	14	10/4	25.5 (3.6)	20 ARMS	11/9	26.65 (5.04)	AN	ARMS > HC P = 0.003
	Fusar-Poli 2011c [62]	fMRI + MRS - C-s	VF	Overt verbal fluency	17	10/7	25.5 (3.6)	24 ARMS	23/1	26.6 (5)	AN	PANSS general ARMS: 21.5 (SD 4.2)
	Fusar-Poli 2011d [63]	fMRI + sMRI – long	WM	N-back	15		.	15 ARMS				
	Fusar-Poli 2010b [64]	fMRI + PET - C-s	WM	N-back	14		.	20 ARMS			AN	
	Allen 2011b [65]	fMRI + PET - C-s	VM	Verbal encoding and recognition	14	9/5	25.7 (4.1)	20 ARMS	10/10	26.30 (5.14)	AN	ARMS 43.55 (13.89)
	Valli 2011 [66]	fMRI + MRS - C-s (overlap with Allen 2011)	VM	Verbal encoding and recognition	16	.	.	22 ARMS	.	.	AN	.
Basel	Smieskova 2011 [67]	fMRI + sMRI - C-s	WM	N-back	20	10/10	26.50 (4.0)	33 (17 ARMS-ST & 16 ARMS-LT)	ARMS-ST: 13/4 ARMS-LT: 11/5	ARMS ST: 25.24 (6.3) ARMS LT 25.06 (2.3)	Y (2)	*

**Abbreviations:** AN, Antipsychotic naives; APS, Treatment with antipsychotics; ARMS, at-risk mental state; C-HR, clinical high-risk; C-HR-NT, C-HR not converted to psychosis; C-HR-T, C-HR converted to psychosis; ChSCZ, C-HRonic Schizophrenia; DMTS, Delayed matching to sample task; Duration ARMS and MRI (yrs), Duration between ARMS identification and MRI scan; EF, executive functioning; ESch, Early Schizophrenia; FEP, first episode of psychosis; GC-HR, G-HR with present pre-psychotic symptoms; HSCT, Hayling sentence completion; M/F, male/female; N, number of individuals; PAL, Paired Associate Learning; PANSS, Positive and negative symptoms (when not other specified, the reported value is TOTAL PANSS SCORE); PROD, for prodromal = at risk stage of schizophrenia - basic symptoms; SC, social cognition; SCZ, schizophrenia patients; SoP, speed of processing; ToM, "Theory of mind"; VL&M, verbal learning and memory; WM, working memory 7 studies reported on clinical follow-up (subjects were followed-up after the fMRI scan, months of follow-up are in brackets): Allen 2012 [46] (24 months), Fusar-Poli 2010a, 2011a [53, 54] (12 months), Shim 2010 [60] (24moths); Brüne 2011 [27] (12 months), Sabb 2010 [56] (6-24 months), Smieskova 2011 [67] (2.88 months by ARMS-ST and 55.44 months by ARMS-LT) 8 studies reported on transition to psychosis (transition rate in brackets): Allen 2012 [46] (17%), Fusar-Poli 2011a, 2010a [53, 54] (13%), Brüne 2011 [27] (10%), Sabb 2010 [56] (7.5%), Gee 2012 [55] (10%), Morey 2005 [57] (20%), Shim 2010 (15.8%) [60];Allen 2011b [65] (15%), Smieskova 2011 [67] (3.3%) Five studies reported on data from SCZ patients treated with antipsychotics (N of treated in brackets): Broome 2009 overlapping with Broome 2010a,b, Benetti 2009 with 10 (7), Brüne 2011 with 22 (22), Choi 2011 with 15 (15), Morey 2005 with 26 (23) and Smieskova 20011 with 21 (8) patients.

* All but one studies reported PANSS (mostly total score), Smieskova 2011 reported SANS total: ARMS ST 21.88 (SD 13.04), ARMS-LT 10.53 (SD 15.20). BPRS total: ARMS ST 40.30 (SD 8.33) ARMS LT 32.31 (SD 6.27).

**Table 4.** Functional Results from Studies of Individuals at Genetic High-Risk for Psychosis

Specification of study		Brain region engaged	Behavioral difference	Differences in brain activation	
Cognitive domain (N)	Task			G-HR vs. HC	G-HR-T vs. G-HR-NT GC-HR vs. G-HR
Verbal memory (6)	Verbal encoding/classif ication and recognition	IFG, SFG, mFG, insula, PL and cerebellum, basal ganglia, thalamus and hippocampus	no differences	***Encoding***: **G-HR** (Val/Val) **< HC** (Met carriers) in IOG. ***Retrieval*: G-HR < HC** in CG, R DLPFC and posterior ParC [13] + *Word classification*: **G-HR **and **C-HR > HC**: R IFG. *Correct recognition*: **G-HR **and** C-HR > HC**: R cerebellum [35]	***Word classification***: **GC-HR > G-HR**: L IPL. *Correct recognition*: **G-HR < GC-HR**: thalamus [13]
	Lexical comprehension + discrimination	STG [42]	no differences	**G-HR < HC**: STG, L supramarginal gyrus, L IFG [42]+ G-HR and **SCZ < HC**: BA44 and BA45 of frontal lobe **G-HR < HC**: BA44-45 in L hemisphere [41]	NA
	Sentence completion	L IFG, medial FG/SFG and L MTG	no differences	Ns [33]	**G-HR-T < G-HR-NT**: ACG. G-HR**-T vs. all** other groups: smaller increases in activation with increasing task difficulty in R lingual gyrus and in TL [34], **HR NP (GC-HR at fu) > HR NN (G-HR at both time points)**: activation over time in L MTG [33]
Working memory (3)	N-back *	ACC, PFC, , basal ganglia, thalamus and cerebellum	SCHIZ < HR < HC performance [36], G-HR < HC: auditory WM task [43]	**G-HR and SCZ > HC**: ParC and Nc accumbens + L ParC and bil cerebellum (***word stimuli***). **G-HR and SCZ < HC**: L ParC and bil cerebellum (*face stimuli* [36]) + **G-HR > HC**: R DLPFC (*2-back*) [43]	NA
	Spatial WM	***Encoding***: occipital cortex, SFG, R precentral gyrus, DLPFC, R VLPFC, caudate, and R thalamus. *Maintenance*: occipital cortex R IPL and L precuneus, and R SFG, DLPFC, and VLPFC R ACC, R insula, and L thalamus. *Retrieva*l: bil IPL and VLPFC, R ACC	G-HR and SCZ < HC: RT	***Encoding***: **G-HR > HC**: R IPL, DLPFC, STG. ***Maintenance*: G-HR > HC**: STG and L MTG, thalamus, and insula *Retrieval*: **G-HR<HC**: R medial FG and L precuneus. [38]	NA
Attention (2)	AX-CPT (variation of the continuous performance)	DLPFC, precentral gyrus, insula, TL, IPL	G-HR < HC in the long delay condition	General task-related activity: **G-HR > HC**: bil DLPFC, precentral gyrus, R SFG and IFG, R SPL and IPL, R medial TG, R insula, R caudate, R claustrum, R cerebellum and L thalamus. Short delay**: G-HR > HC**: activation in DLPFC. Long delay**: G-HR > HC**: L MFG [37].	NA
	Attention	MTG, STG, ITG, SFG, MFG, pre- and postcentral gyri, thalamus, basal ganglia, CG, IPL, SPL, MOG, IOG.	G-HR < HC: RT during the letters task	***Sustained attention task***: **G-HR < HC**: R IPL, SPL, subcallosal G, MOG, MTG, lingual G, parahippocampal G, L VS, precentral and lingual G. **G-HR > HC**: L STG, R MTG. *** Selective attention task*: G-HR < HC**: basal ganglia, R parahippocampal G, precuneus, R insula. **G-HR > HC**: bil MTG, L SFG, supramarginal G, parahippocampal G. ***Dual attention task*: G-HR < HC**: bil IFG, MFG, L IPL, L CG, and hypothalamus [40].	NA
Social cognition (2)	"Theory of mind"	PFC, MTG and STG, precuneus, IPL	HR+Ever were quicker at button pressing than HR+Now & HR ill	**G-HR > C-HR** in R IPL and bil MFG, **G-HR > HR-T**: R cingulate/paracentral lobule [32]	**G-HR-T < G-HR-NT**: R MFG [32]
	Facial emotion recognition	Amygdala, hippocampus, PFC and ParC	NA	***Fearful facial expression***: **G-HR <HC**: activation of L insula, L medial FG, R IFG, R cingulate gyrus, R precentral gyrus and R IPL, R amygdala [45]	NA
Default mode network DMN (2)	Resting state fMRI	mPFC (MFG, SFG), ACC, PCC, IPC, MTG, ITG, STG, Pre, cerebellum	NA	**G-HR vs. HC:** hypoconnectivity in ACC, SFG, PCC, Pre, cerebellum [39]; hyperconnectivity in mPFC [44]	NA

Abbreviations: *: The only one study reported HR vs. FEP or SCZ difference: HR and HC > SCZ: L temporal cortex [36] ACC, anterior cingulate cortex; bil, bilateral; DMN, default mode network; GC-HR, G-HR with present pre-psychotic symptoms; IPL, inferior parietal lobule; MFG, middle frontal gyrus; mFG, medial FG; mPFC, medial prefrontal cortex; MTG, middle temporal gyrus; N, number of studies, NA, not announced; IPC, inferior parietal cortex; ITG, inferior temporal gyrus; OG, occipital gyrus; ParC, parietal cortex; PCC, posterior cingulate cortex; PFC, prefrontal cortex; PL, parietal lobe; Pre, precuneus; SFG, superior frontal gyrus; STG, superior temporal gyrus; TL, temporal lobe; VS, ventral striatum, HR NN, HR asymptomatic at both time points. HR NP, went from asymptomatic to symptomatic [33]

**Table 5.** Functional Results from Studies of Individuals at Clinical High-Risk for Psychosis

Specification of study		Brain region engaged	Behavioral difference	Difference in brain activation		
Cognitive domain (N)	Task			HR vs. HC	HR-T vs. HR-NT	HR vs. FEP or SCHIZ
FUNCTIONAL STUDIES						
Working memory (6)	N-back [51, 58]	ACC, and bil PFC, ParC, basal ganglia, thalamus and cerebellum	No differences	**ARMS < HC: ** L IPL and R angular G ( ***1-back***) [51]. **ARMS < HC: **R insula, L IFG, R IPL, L precuneus and R medial FG, SFG ( ***2-back***) [51] + L SPL and IPL, left precuneus and right postcentral G ( ***2-back vs. 0-back***) [58]. **C-HR > HC **: anterior insula and precentral G ( ***2-back vs. 0-back***) [58]	NA	**ARMS > FEP: ** L IPL and R angular G ( ***1-back***) [51]+ R insula, L IFG, R IPL, L precuneus and R medial SFG ( ***2-back***) [51]
	Spatial delayed-response task [38]; Delayed matching to sample (DMTS) [52]	***Encoding***: OC, SFG, R precentral G, DLPFC MFG, SFG, R VLPFC insula and ACG, bil caudate, and R thalamus. ***Maintenance***: bil occipital cortex R IPL and L precuneus, and R SFG, DLPFC, and bil VLPFC, R ACC, right insula, L thalamus. *** Retrieval/Recognition:*** bil IPL, SPL, precuneus, cuneus, IOG and cerebellar cortex, and VLPFC, R ACC, IFG and insula	SCZ < HC: RT [38]. FEP < HC: correct responses (ARMS not) [52]	**Encoding **: **UHR< HC **: R IPL, DLPFC and L ITG + **UHR > HC ** R STG. **Maintenance **: **UHR < HC **: R parietal areas **+ UHR > HC **: STG, MTG, insula, and ACC. ** Retrieval **: **UHR < HC **: R VLPFC + **UHR > HC **: L DLPFC [38]	NA	***Recognition***: **FEP > ARMS & HC **: bil. IFG and STG, R insula and MTG [52]
	Paired Associate Learning PAL (visuospatial) [50, 54]	Cerebellum, MOG, precuneus, MTG, bil. fusiform and precentral G, R insula and MFG, L thalamus, putamen	ARMS > HC: latency in responses during the hardest level of the task [54], FEP < HC: accuracy during the intermediate and hardest level of the task [50]	**ARMS < HC **: L precuneus/occipital G, L SPL and R MTG [54]. ***Intermediate level***: **ARMS < HC **: R cerebellum, precuneus and medial FG / SFG. ***Hard level***: **ARMS < HC ** medial FG, SFG and right precuneus [50]	NA	***Easy level***: **FEP < HC ** = ARMS medial FG and ACG. *** Intermediate level***: **HC > ARMS > FEP ** R cerebellum, precuneus and medial FG / SFG. *** Hard level***: **ARMS > FEP ** medial FG, SFG and right precuneus [50].
Social cognition (5)	"Theory of mind" [27, 32]	mPFC, ACC, PCC, precuneus, MTG, STG, TPJ	No differences [27], G-HR were quicker at button pressing than GC-HR [32]	**C-HR < HC **: medial FG and PCG + C-HR ** > HC **: L IFG, TPJ, STG/MTG and PCG, R precuneus [27]	**HR-T < HR-NT **: R MFG [32]	**C-HR > SCHIZ **: L IFG, TPJ, STG/MTG billy + **C-HR < SCHIZ **: R precuneus [27]
	Emotion processing task [55, ,58 59]	Amygdala, hippocampus, prefrontal and parietal cortices	No differences [,^55^ ,^58^ ^59^]	**C-HR > HC: ** amygdala + **C-HR < HC **: VLPFC [55]; ***Negative emotions***: **C-HR < HC **: R insula, L medial STG and the R MOL [58]. **C-HR > HC **: R PCG and MTG [58] + ***Neutral vs emotional faces:*** L IFG, thalamus, cuneus, hippocampus and SFG [59]	NA	NA
Verbal fluency (3)	Overt verbal fluency [46, 51, 53]	L SFG, precentral G and IFG, L insula and thalamus bilaterally, L middle FG, left pre- and postcentral G, frontal operculum and insula, SFG, R insula, caudate and brainstem.	No differences [46, 51, 53]	**ARMS < HC **: the ACG [53] and IFG by *easy condition * [51] to middle FG and precentral G by ***hard condition*** [51]; **ARMS > HC **: the L IFG [53] and R MFG and SFG [46] and L anterior insula [51]	**UHR-T > UHR-NT **: L SFG, MFG, brainstem, L hippocampus + **UHR-T > HR-NT **: in brainstem (18F)-DOPA ki [46]	**ARMS > FEP ** L IFG ( ***easy condition***) + middle FG and precentral G ( ***hard condition***). **FEP > ARMS ** L anterior insula [51]
Verbal memory (2)	Hayling sentence completion task (HSCT) [48]	L SFG, VL IFG, MFG, Insula, STG, ITG, MTG, cuneus, basal ganglia, thalamus, hippocampus	No differences [48]	ARMS > HC: ***Recognition during false alarms vs correct recognition***: hippocampus + ***Response initiation and suppression***: R caudate and bil ACC [48]	NA	NA
	Verbal encoding and recognition [47]	L IFG, MFG and precentral G, IPL, fusiform G	ARMS lower recognition accuracy [47]	***Encoding***: **ARMS < HC **: bil medial FG, L MFG, L middle parahippocampal G [47]	NA	NA
Executive functioning (1)	Random movement generation task [49]	L insula, pre- and postcentral G and L medial FG, cerebellum, L PL and L supplementary motor area	Movements to the right: HC > FEP > ARMS [49]	**ARMS < HC: ** L insula, L postcentral G and L IPL [49]	NA	**ARMS > FEP: ** L insula, L postcentral G and L IPL [49]
Attention (1)	Visual oddball continuous performance task [57]	SFG, MFG, CG, MTG, STG, ITG, pre- and postcentral gyri, thalamus, basal ganglia, IPL, SPL, MOG, IOG	Performance: HC > UHR > FEP > ChSCZ. Impaired target discrimination in UHR and FEP relative to HC [57]	***Target vs. novel differential activation***: **UHR < HC **: ACG, MFG and IFG [57]	NA	***Target vs. novel differential activation***: **UHR > ESch > ChSCZ **: ACG, MFG and IFG [57]
Language processing (executive and semantic processing) (1)	Naturalistic discourse processing paradigm [56]	bil inferior temporal lobe, MTG, hippocampus, IFG, and precentral G	No differences [56]	**Reasoning condition **: C-HR > HC: L IFG, ACG, L ITG and MTG and occipital cortex [56]	***Reasoning condition***: C-HR-T > C-HR-NT: TL, frontal operculum, L precentral G, caudate and others striatal regions [56]	NA
Default mode network (DMN) (1)	rs fMRI	mPFC (MFG, SFG), ACC, PCC, IPC, ITG, STG, MTG, Pre, cerebellum	NA	**UHR vs. HC: **hyperconnectivity between PCC and ACC, mPFC, Pcu, LPC [60]	NA	NA
MUTIMODAL STUDIES						
Working memory (3)	N-back task [63, 64, 67]	ACC, and bil PFC, ParC, basal ganglia, thalamus and cerebellum	FEP and ARMS-ST > HC and ARMS LT: RTs [67]	**ARMS < HC **: R MFG, L medial FG, L SPL [64], L MFG (no more different after GMV as covariate in VBM-fMRI analysis), supramarginal G, IPL [63]. **ARMSST < HC**: bil SPL, R IPL, L SFG [67]. **ARMS at f-u > ARMS at bl:** activation in A ACG, parahippocampal G + no VBM changes [63]	*ARMS-ST < ARMS-LT : R IFG, insula and L SFG, insula and bil precuneus [67]	**FEP < ARMS-LT **: bil IFGs and insula, L SFG and MFG [67].
Verbal fluency (2)	Overt verbal fluency [61, 62]	L SFG, precentral G and IFG, L insula and thalamus bil, L middle FG, left pre- and postcentral G, frontal operculum and insula, SFG, R insula, caudate and brainstem	No differences [61, 62]	ARMS >HC: L IFG and R MFG [61, 62] MRS: ARMS < HC: Glu levels in L thalamus and L hippocampus [62]	NA	NA
Verbal memory (2)	Verbal encoding and recognition [65, 66]	L insula and MTG, STG and L IFG, bil MFG and SFG	ARMS > HC: false alarm responses + more OLD responses for Novel words (i.e. False Alarms) [65].	***Encoding***: **ARMS < HC **: L parahippocampal G [65, 66]. *** Correct recognition***: no diff in activation [66] negative correlation between activation and ki for F-Dopa in subiculum and bil hippocampus in the limbic striatum in HC but not in ARMS [65]	NA	NA

**Abbreviations**: ACC, anterior cingulate cortex; ARMS, at-risk mental state; ChSCZ, C-HRonic schizophrenia; G, gyrus; GC-HR, G-HR with present pre-psychotic symptoms; IPL, inferior parietal gyrus; ITG, inferior temporal gyrus; L, left, LPC, lateral prefrontal cortex; mPFC, medial pefrontal cortex; MTG, middle temporal gyrus; N, number of studies; NA, not accounced. OC, occipital cortex; ParC, parietal cortex; PCC, posterior cingulate cortex; Pcu, precuneus; R, right, rs fMRI, resting state fMRI; SC, social cognition; SFG, superior frontal gyrus, SoP, speed of processing; STG, superior temporal gyrus; TL, temporal lobe; TPJ, temporo-parietal junction; VL IFG, ventrolatreral inferior frontal gyrus;

* **ARMS-ST** < **ARMS-LT**, no difference ARMS-ST vs. ARMS-NT, difference associated with the duration of the ARMS status, no with transition to psychosis
